# Effects of Ageing on Aortic Circulation During Atrial Fibrillation; a Numerical Study on Different Aortic Morphologies

**DOI:** 10.1007/s10439-021-02744-9

**Published:** 2021-03-02

**Authors:** Amin Deyranlou, Christopher A. Miller, Alistair Revell, Amir Keshmiri

**Affiliations:** 1 Department of Mechanical, Aerospace and Civil Engineering (MACE), The University of Manchester, Manchester, M13 9PL UK; 2 Division of Cardiovascular Sciences, School of Medical Sciences, Faculty of Biology, Medicine and Health, University of Manchester, Manchester Academic Health Science Centre, Oxford Road, Manchester, M13 9PL UK; 3 Manchester University NHS Foundation Trust, Manchester Academic Health Science Centre, Southmoor Road, Wythenshawe, Manchester M13 9PL UK; 4 Wellcome Centre for Cell-Matrix Research, Division of Cell-Matrix Biology and Regenerative Medicine, School of Biology, Faculty of Biology, Medicine and Health, University of Manchester, Manchester Academic Health Science Centre, Oxford Road, Manchester, M13 9PL UK

**Keywords:** Aorta, Atrial fibrillation, Ageing, Cardiovascular diseases, Haemodynamic metrics, Computational fluid dynamics

## Abstract

Atrial fibrillation (AF) can alter intra-cardiac flow and cardiac output that subsequently affects aortic flow circulation. These changes may become more significant where they occur concomitantly with ageing. Aortic ageing is accompanied with morphological changes such as dilation, lengthening, and arch unfolding. While the recognition of AF mechanism has been the subject of numerous studies, less focus has been devoted to the aortic circulation during the AF and there is a lack of such investigation at different ages. The current work aims to address the present gap. First, we analyse aortic flow distribution in three configurations, which attribute to young, middle and old people, using geometries constructed *via* clinical data. We then introduce two transient inlet flow conditions representative of key AF-associated defects. Results demonstrate that both AF and ageing negatively affect flow circulation. The main consequence of concomitant occurrence is enhancement of endothelial cell activation potential (ECAP) throughout the vascular domain, mainly at aortic arch and descending thoracic aorta, which is consistent with some clinical observations. The outcome of the current study suggests that AF exacerbates the vascular defects occurred due to the ageing, which increases the possibility of cardiovascular diseases *per se*.

**Supplementary Information:**

The online version contains supplementary material available at (10.1007/s10439-021-02744-9).

## Introduction

Atrial Fibrillation (AF) is the most frequent arrhythmia, which prompts irregular heartbeats. The increasing rate of AF^40^ and associated financial burden^11^,^68^ have combined to create an ever growing issue for the healthcare system. AF is generally accompanied by electrophysiological and structural changes of the heart,^70^ which mainly causes left atrial remodelling, absence of active contraction, shortage in ventricular function, and high frequency fibrillation (HFF). Clinical studies classify AF as a complex disease with an increasing risk of other cardiovascular diseases (CVDs) such as myocardial infarction, heart failure, valvular heart disease, and stroke.^29^,^40^ Amongst these, stroke incidence is one of the most frequent side effects of AF.^15^,^33^,^46^ In fact, it has been hypothesised that the primary reason for stroke is due to the clot formation inside the left atrium, and its subsequent movement towards the cerebral arteries.^43^ Aside from the intra-atrial embolism, intravascular plaque formation^5^ and atherosclerosis^71^ represent another significant consequence of AF occurrence that might be worsened as a result of ageing.^38^ While to date there have been a large number of studies on the association of AF and the aforementioned dysfunctions, the outcomes have sometimes been contradictory, specifically in stroke occurrence, and as such a number of important questions remain unanswered.

The study of haemodynamics within large vessels or through microcirculations is utilised as a procedure to better understand AF effects on heart, brain, and the vasculatures. In addition to *in-vivo*/*vitro*, and *ex-vitro* studies, computer simulation offers the opportunity to isolate parameters and investigate their standalone impact on the system. Examples of such endeavours with particular focus on AF, include assessment of its impact on coronary^64^ and cerebral circulation,^62^,^65^ changes in valvular function,^63^,^66^ intra-atrial flow structure,^35^,^42^,^48^,^56^ and likelihood of thrombus formation inside the atrial appendage.^7^,^27^ Despite these efforts, less attention has been given to the haemodynamics of aortic flow during AF.^14^,^19^

A recent study by the present authors reported an investigation into the blood flow in aortic circulation during AF.^19^ The results suggested that, because of decrease in left ventricular outflow, the endothelial cell activation potential (ECAP) increases that leads to raising of thrombo-prone regions at aortic arch and proximal descending thoracic aorta (PDTA). The previous study was focused on a single case, while from a clinical point of view a significant variation of aortic morphology is anticipated, for a population with varying age, gender, ethnicity and disease. From the haemodynamic perspective, it is clear that even minor geometric changes can lead to a significant variation in flow distribution throughout the aorta and its branches and, as such, the present work is one of the very few studies, which aims to evaluate aortic circulations during a concomitant occurrence of AF and age-related geometric variations. We present a computational parametric study to explore aortic haemodynamics at different age groups during both normal and AF cardiac functions.

## Materials and Methods

### Ageing and Geometrical Changes

Aortic morphology alters between different people due to the gender, ethnicity, and physical factors such as body surface area and body mass index. Additionally, each individual can experience structural changes in the aorta over its lifetime mainly due to ageing and diseases. Age is recognised as an autonomous risk factor for various CVDs. It is associated to a reduction in endothelial cell functionality, genesis of superfluous connective tissue and changes in arterial pressure, which cause vascular wall stiffening, dilation, and lengthening.^3^

Commonly obtained metrics characterising the morphology of an aorta include: diameter, curvilinear length between different sections, height, width, angulation and curvature of the arch, and tortuosity.^8^,^16^,^61^ Another factor is the configuration of the supra-aortic trunk (SAT). During the embryonic period and in course of aortic arch evolution, SAT develops different configurations. The ‘standard type’, the most common configuration, has a separate origin of brachiocephalic artery (BCA), left common carotid artery (LCCA), and left subclavian artery (LSCA). In a review article by Popieluszko *et al*.^58^ on over 23,000 cases throughout the globe, between 65 and 87% of the population have a standard aortic arch, while the remaining develop other SAT configurations in which bovine arch—common trunk of BCA and LCCA—is the most frequent anomalous type.

In this study a comprehensive meta-analysis has been carried out on geometrical specifications of aorta. To the best of our knowledge, this is the first such endeavour that has been performed in this context, incorporating more than 40,000 cases of healthy populations, based on the data from 28 original and review articles. The inclusion criteria for the incorporated data were healthy individuals without any overt CVD or associated risk factors with a standard type of SAT. Access to the full list of the articles is provided in the Supplementary Data.

**Figure 1:**
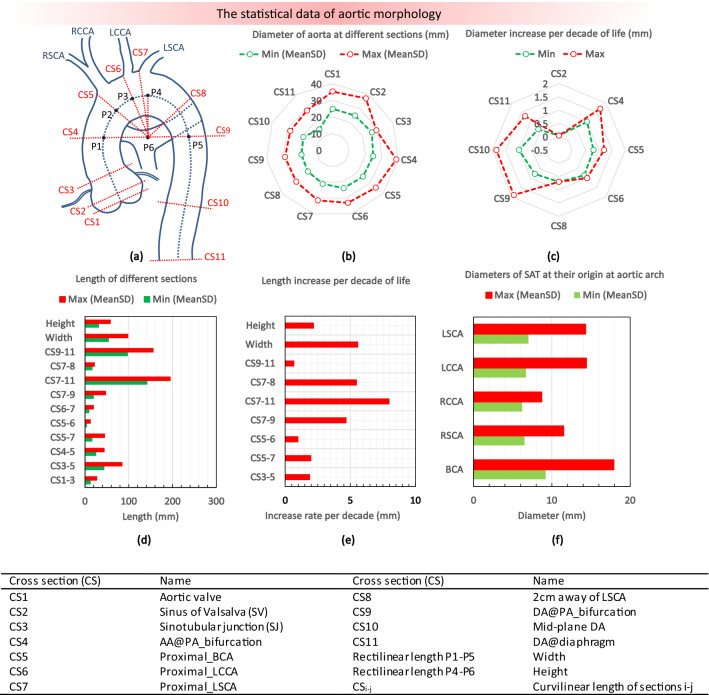
The statistical data of aortic morphology; (a) a schematic of an aorta with a normal SAT, (b) min and max values of the mean diameter at eleven different sections, (c) diameter increase rate per decade of life, (d) curvilinear length between different sections, (e) length increase per decade of life, (f) min and max values of the mean SAT diameters at the origin, with an increase range of 0.14–0.3 mm per decade of life.

A summary of the analysis is presented in Fig. 1 for the diameter and recti/curvilinear length of aorta at defined sections. The results are shown as the average minimum and maximum of the population, who mainly falls between 20 and 80 years old, irrespective of the age, gender, and ethnicity. Considering the rate of diameter increase per decade of life, at sinus of Valsalva (CS2) a wider range between 0.04 mm and 1 mm was reported,^18^,^44^ while most of the studies, as documented in the Supplementary Data suggested a slight variation at the aortic root. Furthermore, at the ascending (CS3–CS5) and descending (CS9–CS11) regions, a higher diameter growth rate occurs (Fig. 1c). At the SAT branches, the growth rate is between 0.14 and 0.3 mm per decade of life (Fig. 1f), which is much smaller than the main aortic conduit. Finally, considering the length of different segments, the largest growth is suggested for the region located between CS7 and CS9, which leads to the aortic arch widening and unfolding.

**Figure 2:**
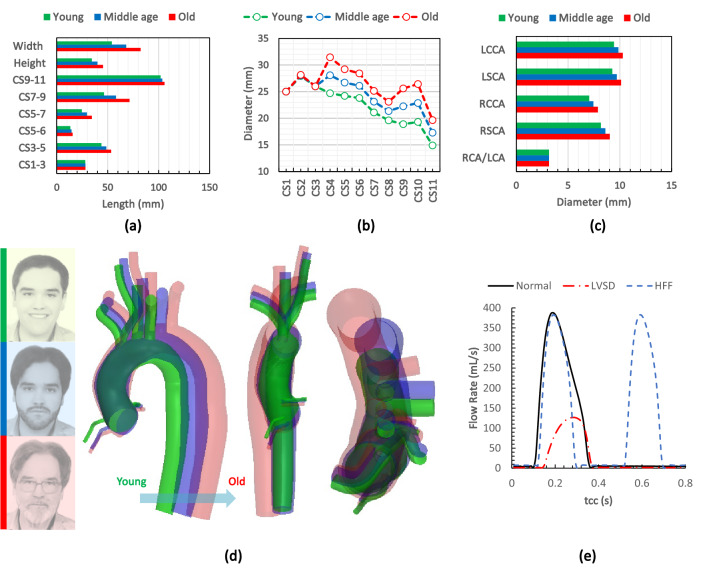
Geometrical specifications of young, middle age and old groups; (a) curvilinear length between different sections, (b) diameter of the aortic conduit at different sections, (c) diameter of the coronaries and SAT at their origin, (d) constructed aortic geometries for different age groups; (e) flow rates at the inlet, *Q*(*t*).

Using these clinical measurements, characteristic geometries representing three age ranges (young, middle, old) have been constructed for computational analysis in the present work, as described in Fig. 2. Starting from the minimum average values for each segment and based on the mean values of growth per decade of life, new datasets are estimated. Initially, the groups were categorised based on the decade of life as 20–30, 30–40, 40–50, 50–60, 60–70, and 70–80. Then the geometries were constructed for the young, middle age, and old groups, which fall between 20–30, 40–60, and 70–80, respectively. All three geometries are constructed based on the centre-points and path-lines of the 31-year old healthy aorta in our previous study.^19^ Finally, the reference geometry was modified for each age group using the CAD software, SolidWorks 2017 (SP 2.0) according to the parametric variations identified in Fig. 1. Note that the angle between BCA and LSCA of the constructed aortic models varies between 31° and 34°, and is not significantly correlated with age; which is in agreement with the clinical findings.^8^,^28^ Moreover, age-associated dilation and arch unfolding are correctly addressed in the created geometries. Worth mentioning that, the constructed models represent a population with a low tortuosity at the descending thoracic aorta (DTA).

### Ageing and Elevated Pressure

Arterial wall structure changes continuously due to ageing and disease onset. Independently, ageing causes arterial wall stiffening, specifically in large central arteries.^3^ Changes in viscoelastic property of the arterial wall, increase in systolic/pulse pressure, and changes in pulse wave reflection, all suggest changes in arterial compliance. Moreover, ageing increases mean arterial pressure,^55^ while the normal cardiac output can be preserved in healthy populations.^31^

Therefore, to incorporate age-associated elevated pressure, three different data sets were calculated for the resistance and compliance of three-element Windkessel (RCR WK) model. In order to calculate each data set, the mean blood pressure was assumed to increase within the normal range, while the mean flow rate at the aortic root was assumed to be constant for all the age groups.^55^ The selected approach induces a correct elevated mean pressure within a cardiac cycle. Furthermore, the parameters are tuned to mimic a normal perfusion through different branches.^4^ Note that, in AF, the coronary perfusion, particularly during diastole reduces, owing to the changes in myocardial resistance component of the coronary artery.^41^ However, given the small portion of flow going through the coronary arteries, the AF associated changes in the WK model of coronary were neglected. Moreover, since there is no direct relation between AF and changes in RCR parameters of the downstream aortic branches, it was assumed that the RCR parameters are not affected by AF. Table 1 presents the chosen values for different age groups. More details are provided in the Supplementary Data.

**Table 1:** RCR WK parameters for each outlet.

Age group	Young			Middle age			Old		
Parameter	*R*_p_× 10^−8^ (Pa s/m^3^)	*R*_d_× 10^−8^ (Pa s/m^3^)	*C *× 10^10^ (m^3^/Pa)	*R*_p_× 10^−8^ (Pa s/m^3^)	*R*_d_× 10^−8^ (Pa s/m^3^)	*C *× 10^10^ (m^3^/Pa)	*R*_p_ × 10^−8^ (Pa s/m^3^)	*R*_d_ × 10^−8^ (Pa s/m^3^)	*C *× 10^10^ (m^3^/Pa)
RCA	10.1	102.3	1.47	11.0	110.8	1.36	11.8	119.3	1.3
LCA	3.9	39.8	3.8	4.26	43.1	3.5	4.6	46.4	3.2
SAT	1.9	19.1	7.9	2.0	20.7	7.3	2.2	22.3	6.8
DTA	0.2	2.2	68.4	0.2	2.4	63.1	0.3	2.6	58.6

### Ageing, AF, and Present Test Cases

In our previous study,^19^ four primary AF-associated defects were modelled, *via* introduction of four different inlet boundary conditions applied to the reference geometry. These were lack of atrial kick, left atrial remodelling, left ventricular systolic dysfunction (LVSD), and HFF. The waveform corresponding to each AF-associated defect was obtained from the left heart lumped model, initially proposed by Simaan *et al*.^67^ and modified in our previous study.^19^ Aortic flow patterns during both severe LVSD and HFF were observed to be significantly different to normal flow,^19^ and as such we focus on these cases here. Then, the obtained flow waveforms are directly applied to the inlet of aorta as shown in Fig. 2e.

In the present study, for each age category (Fig. 2d), three simulations were performed to compare the normal flow rate to the severe LVSD and HFF condition of 150 bpm. For more details about each AF associated defect, please refer to our previous work.^19^

### Governing Equations

In this study, the blood flow is considered to be a laminar, incompressible, homogenous and non-Newtonian fluid. In order to simulate the flow, the continuity equation along with the momentum equations are invoked as follows:

1 $$\frac{\partial \rho }{\partial t} + \frac{\partial }{{\partial x_{i} }}\left( {\rho u_{i} } \right) = 0$$

2 $$\frac{{\partial \rho u_{j} }}{\partial t} + \frac{\partial }{{\partial x_{k} }}\left( {\rho u_{k} u_{j} } \right) = \frac{{\partial \sigma_{{f}_{ij}} }}{{\partial x_{i} }}$$

In which $\rho$ is the blood density, equal to 1060 kg/m^3^, $u_{i}$ denotes fluid velocity components, $x_{i}$ is the coordinate system, and the stress tensor, $\sigma_{{f}_{ij}}$, is defined as follows:

3 $$\sigma_{{f}_{ij}} = - p\delta_{ij} + \mu \left( {\frac{{\partial u_{i} }}{{\partial x_{j} }} + \frac{{\partial u_{j} }}{{\partial x_{i} }}} \right)$$

where *p* is pressure, $\delta_{ij}$ is the Kronecker delta, and $\mu$ is dynamic viscosity. In order to account for blood shear thinning effects, the dynamic viscosity is defined based on the Carreau–Yasuda^73^ model as follows:

4 $$\mu = \mu_{\infty } + \left( {\mu_{0} - \mu_{\infty } } \right)\left( {1 + \left( {\lambda \dot{\gamma }_{ij} } \right)^{a} } \right)^{{\frac{n - 1}{a}}}$$

In Eq. (4) $\mu_{0}$ is low shear viscosity and is set to 0.16 Pa s, $\mu_{\infty }$ is high shear viscosity and is taken as 0.0035 Pa s, $\lambda$ denotes time constant equal to 8.2 s, *n* is power-law index that is equal to 0.2128, and *a* is Yasuda exponent, which is equal to 0.64.^10^ Also, $\dot{\gamma }$ is the shear strain rate, which is defined as:

5 $$\dot{\gamma }_{ij} = \sqrt {\frac{{\partial u_{i} }}{{\partial x_{j} }}\left( {\frac{{\partial u_{i} }}{{\partial x_{j} }} + \frac{{\partial u_{j} }}{{\partial x_{i} }}} \right)}$$

Furthermore, it was shown that, the plasma viscosity slightly increases by age for low shear rates below 50 s^−1^.^9^ Thus, in the current work and given the flow regime, similar values are used for the blood properties of different age groups.

### Boundary Conditions

The model comprises one inlet and seven outlets including right coronary artery (RCA), left coronary artery (LCA), right subclavian artery (RSCA), right common carotid artery (RCCA), LCCA, LSCA and DTA. For the inlet, a parabolic velocity profile, $u\left( {r,t} \right)$ was imposed as defined by Eq. (6), which reproduces more accurate inlet in the absence of patient specific velocity profile^74^:

6 $$u\left( {r,t} \right) = 2\left( {\frac{Q\left( t \right)}{A}} \right)\left( {1 - \left( {\frac{r}{R}} \right)^{2} } \right)$$

where $Q\left( t \right)$ is a time-variant flow rate, which is directly obtained from the left heart model described earlier, *A* is the cross-sectional area of the inlet, *R* denotes the radius of the inlet, and *r* is the radial coordinate with respect to the centre of inlet. At the outlets, RCR WK model^39^,^49^,^57^ was prescribed to mimic arterial compliance and upstream pressure. To incorporate the ageing effects, different RCR values were used to consider the relative changes of arterial stiffness with age (Table 1). Furthermore, no-slip conditions were imposed to the vessel walls and the aorta was assumed to be rigid; the latter assumption is a rational trade-off of accuracy, data availability, and the computational cost.^12^

### Numerical Method

The simulations were carried out using ANSYS-CFX 19.2, using the finite volume method. The advection terms were discretised using high resolution scheme.^2^ Moreover, a second order backward Euler scheme was invoked to discretise the time derivative. The convergence criteria for the simulation are based on root mean square (RMS) of residuals of mass and momentum equations and are set to 10^−6^.

To implement RCR WK model, the relevant ordinary differential equations are discretised implicitly using the first order backward Euler method, and applied as the pressure outlets to the domain. Furthermore, at the inlet, a Fourier series with eight harmonics was fitted to the data obtained from 4D PC-MRI/lumped heart model, using the least square method; and applied to the intel.

To obtain a converged solution, which is independent of grid size, four different meshes were examined. The computational domain consists of tetrahedral elements, which are accompanied with five prism layers to capture the near wall effects more accurately. Mesh sensitivity analyses demonstrated that a computational domain with around 3.2–4.5 million elements—the given range shows the chosen grid element numbers for the young, middle age and old aortas—is fine enough to capture all the flow features precisely (refer to the Supplementary Data). Furthermore, for a stable and time-independent solution, timestep size of 0.125 ms was chosen and the simulation was performed for four cardiac cycles to obtain a fully converged temporal solution.

## Results

### Validation

**Figure 3:**
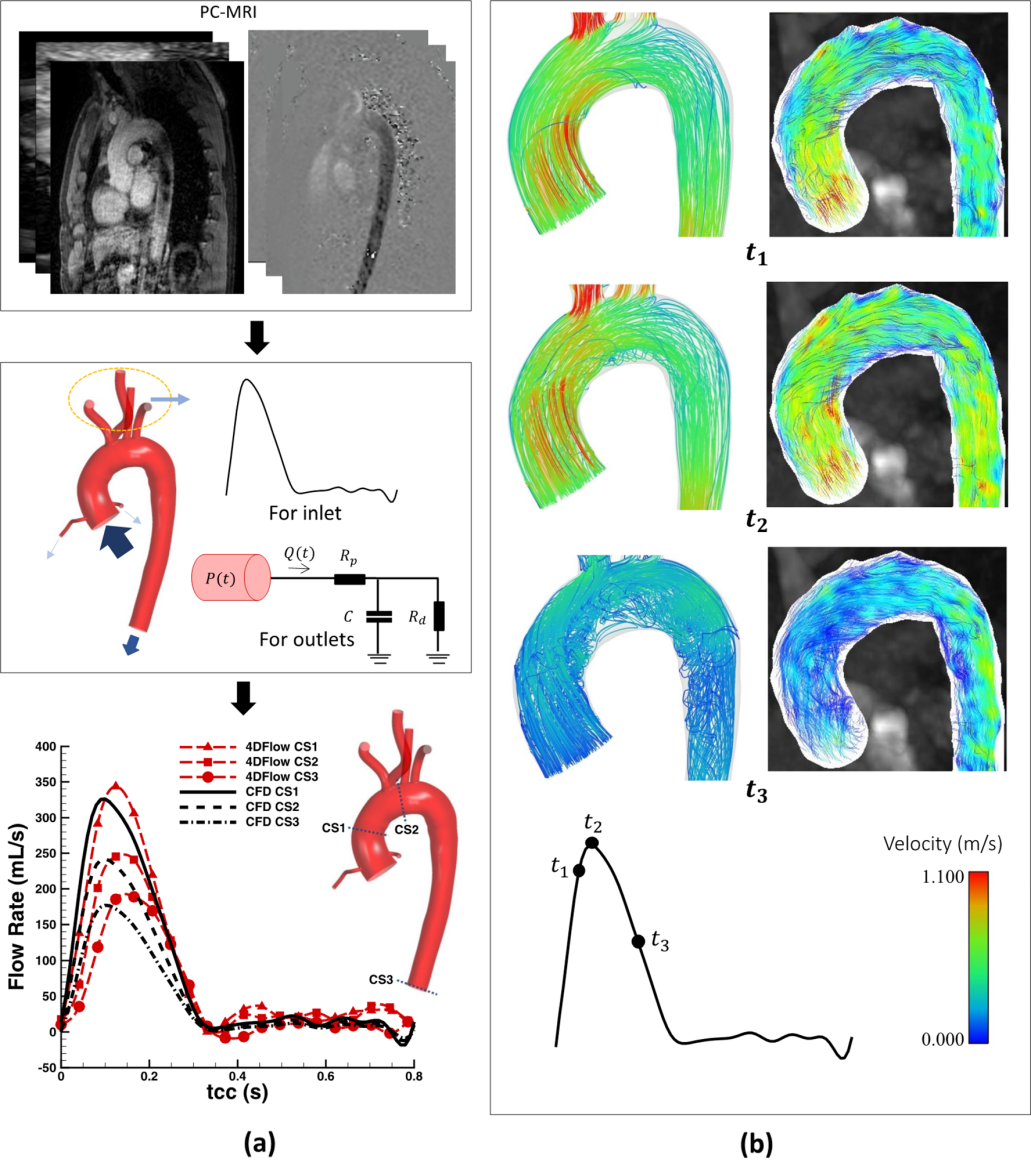
Validation of the numerical code against the PC-MRI data of the 31-year-old healthy volunteer^19^; (a) the flow rates are depicted at three different cross sections (CSs) in a cardiac cycle (*t*_cc_). CS1 is before BCA, CS2 is between BCA and LCCA and CS3 is located at DDTA, (b) a qualitative comparison of streamlines at three time points at the AA, aortic arch, and PDTA.

The validation was carried out by comparing the CFD results with the PC-MRI data of the 31-year-old healthy subject.^19^ For this purpose, flow rates at three different cross sections at ascending aorta (AA), aortic arch between BCA and LCCA and the DTA are compared and presented in Fig. 3a. The numerical results show good agreement with the PC-MRI data, although the CFD tends to slightly underpredict the peak for the flow rates with an earlier occurrence comparing to the MRI data. The small discrepancies are believed to be due to the rigid wall assumption, while for the MRI data, the chosen value for the velocity encoding (VENC)—200 cm/s—settings and associated noises are potential sources of uncertainty. Furthermore, Fig. 3b demonstrates a qualitative comparison of flow streamlines between the phase data and the CFD results at three timepoints at the AA, aortic arch, and PDTA. In addition to the general similarities, both the MRI and CFD suggest occurrence of a helical flow at the chosen regions, which has been widely reported.^36^,^60^

### Flow Structure

**Figure 4:**
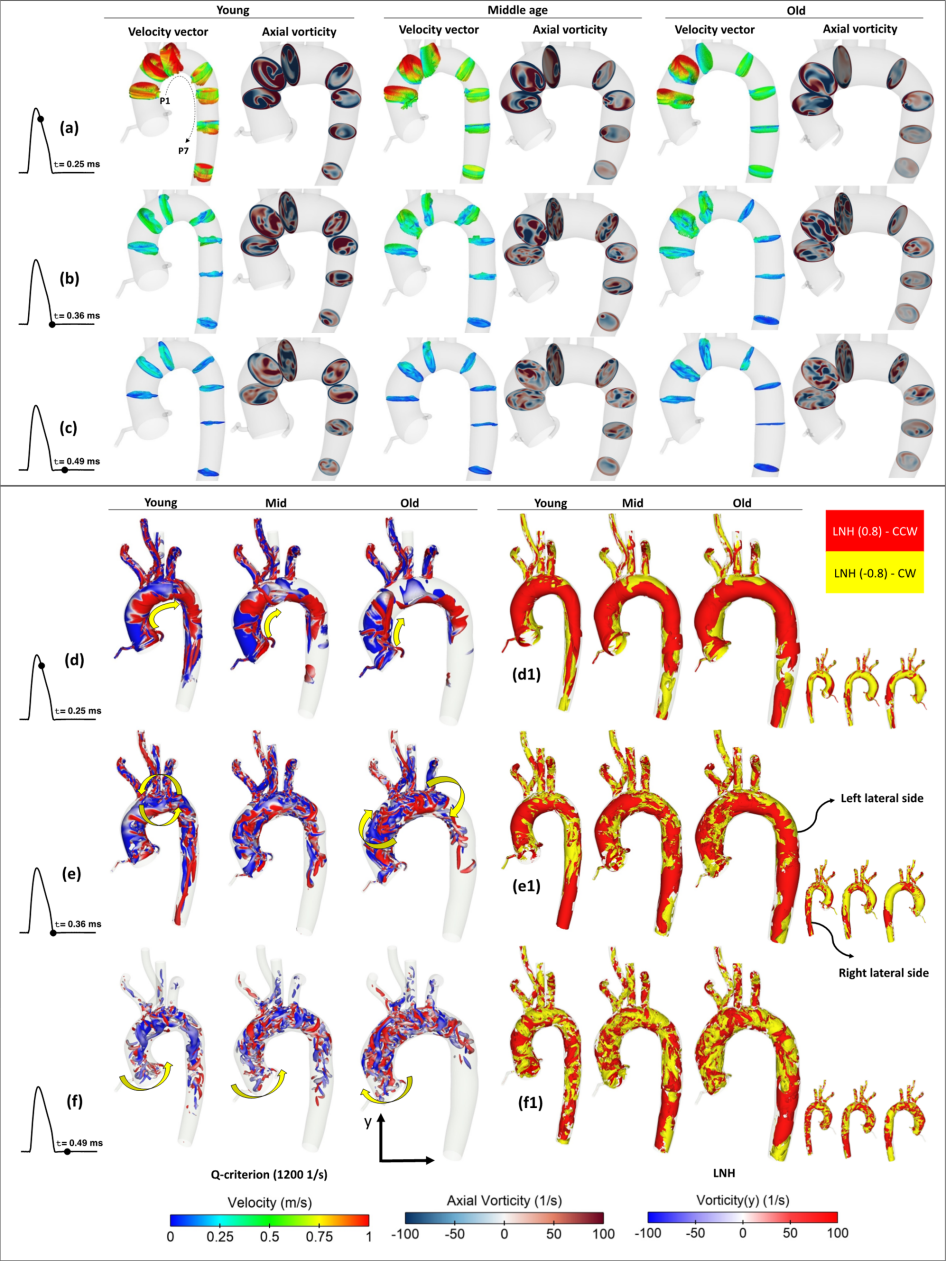
Velocity vector and axial vorticity at three different points in a cardiac cycle (a)–(c), and seven cross sections for young, middle age, and old groups; iso-surface of vorticity, Q-criterion, 1200 s^−1^ (d)–(f), and LNH (d1)–(f1) at three timepoints in a cardiac cycle.

Presenting the overall flow field helps to derive a global understanding of the flow. Such insight is often only possible *via* the production of animations, which chart the evolution of the flow and associated structures throughout the cardiac cycle. Figure 4 provides an example, where snapshots of the aortic flow are included for the young, middle, and old age group geometries at three timepoints in a cardiac cycle.

**Figure 5:**
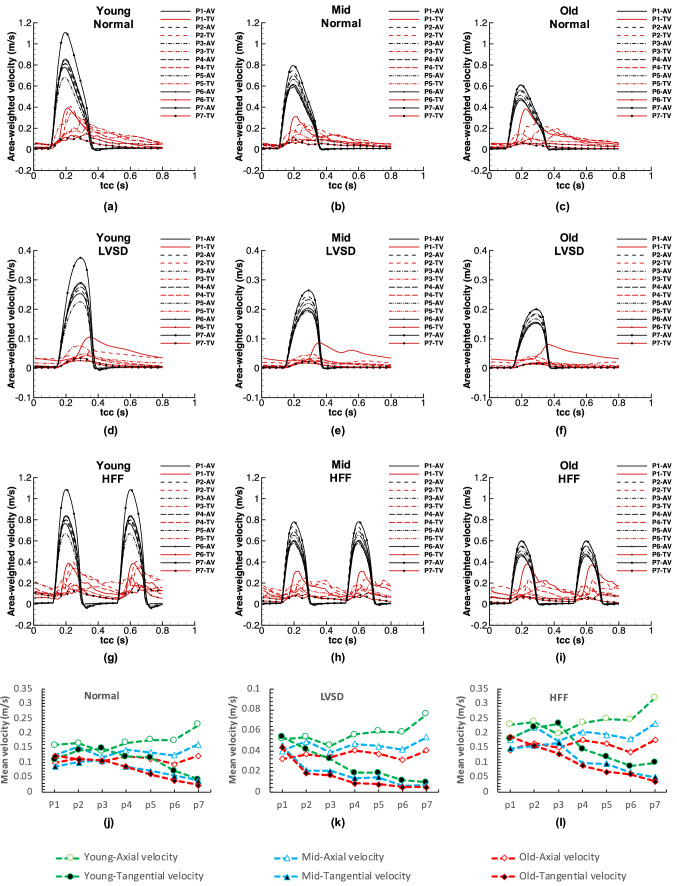
(a)–(i) Area weighted axial velocity (AV) and tangential velocity (TV) components over a cardiac cycle, (j)–(l) area-weighted, time-averaged AV and TV components. The results are shown at seven planes (Pi) in Fig. 4 for the young, middle age, and old groups during the normal, LVSD, and HFF conditions. For the LVSD cases, (d)–(f), the *y*-axis range is modified to clearly present the variations.

In Figs. 4a–4c, the velocity vectors and contours of vorticity are presented. In the context of this paper, the flow direction perpendicular to a plane can be attributed as a primary flow, while an in-plane flow is similar to a secondary flow.^36^,^60^ The results are displayed in three timepoints in a cardiac cycle and seven cross sections corresponding to the CS4, 5, 6, 8, 9,10, and 11 of Fig. 1a. The results show that during the flow acceleration, the axial velocity (AV) is the dominant velocity component, while as it approaches the systolic peak and during the deceleration, the tangential component becomes more significant as confirmed in Figs. 5a–5c. Figure 4a demonstrates that during the systolic peak and flow deceleration, the possibility of flow reversal specifically at the AA and aortic arch increases as confirmed through *in-vivo* data,^47^,^54^ and the occurrence becomes more significant by ageing, which is in agreement with the *in-vivo* observations of Bogren *et al*.^6^ Furthermore, the axial vorticity reveals that for all the groups the dominance of vortex core regions is higher during the systolic peak and late systole compared to early diastole.

Figures 4d–4f show iso surfaces of vorticity (Q-criterion, 1200 s^−1^)^34^ coloured by their rotational direction around the vertical axis and annotations are included to help describe the 3D transient flow field (https://tinyurl.com/Q1200). Furthermore, the relative directions of velocity and vorticity, which are defined as localised normalised helicity (LNH), are shown in Figs. 4d1–4f1 to get a better intuition regarding the flow field changes. Based on the concept of helicity, which was initially introduced by Moffat,^51^ the helicity in aortic flow can be defined as follows:

7 $$H\left( t \right) = \varvec{u}\left( {\varvec{x},t} \right) \cdot\varvec{\omega}\left( {\varvec{x},t} \right)$$

where, $\varvec{u}\left( {\varvec{x},t} \right)$ and $\varvec{\omega}\left( {\varvec{x},t} \right)$ are the velocity and vorticity vectors, respectively. Thus, the LNH can be defined as follows:

8 $$LNH = \frac{{\varvec{u}\left( {\varvec{x},t} \right) \cdot\varvec{\omega}\left( {\varvec{x},t} \right)}}{{\left| {\varvec{u}\left( {\varvec{x},t} \right)} \right|\left| {\varvec{\omega}\left( {\varvec{x},t} \right)} \right|}}$$

Moreover, to analyse the helicity and its regional magnitude, the ‘time-averaged helicity’, $H_{1}$, and ‘helicity intensity’, $H_{2}$, inside a 3D domain with a volume of *V* are introduced as follows^52^:

9 $$H_{1} = \frac{1}{{t_{cc} V}}\smallint \smallint_{V} H\left( t \right)dVdt$$

10 $$H_{2} = \frac{1}{{t_{cc} V}}\smallint \smallint_{V} \left| {H\left( t \right)} \right|dVdt$$

As shown in Fig. 4d, displaying a snapshot just after peak systole, the extent to which flow travels along the aorta varies significantly for the different age groups. Since we assume a similar flow rate for all ages, and since the aortic arch is shown to both distend and widen with age (See Fig. 2d), it follows due to continuity that a given mass flow will penetrate a shorter distance through the vessel in a fixed time. The aortic flow is characterised by highly rotational flow fields, which as shown in Fig. 4e, vary considerably with age group. In the young group, high velocity flow at the top of the arch, proximal to the SAT, induces tight circular motion in the horizontal plane, which in turn promotes a strong helical motion downstream. In contrast, the lower velocity flow, observed by this stage in the old group, which has penetrated a lesser distance along the aorta, gives rise to a larger and slower bulk rotation in the sagittal plane. The flow at this stage for the middle age group is somewhat of a combination of these two effects. In mid diastole, when residual vorticity effects dominate in the absence of a strong streamwise flow, the previously observed differences in the flow field lead to quite different bulk flow. As shown in Fig. 4f, the flow is observed to rotate counter-clockwise (CCW) in the AA for young and middle age groups, while the same region is undergoing a clockwise (CW) rotation for the old age group (refer to the supplementary Data, video 1). While minor deviations from the geometries and conditions tested here can be expected to influence these findings, it is nevertheless significant to underline the impact of age-related morphological variation on bulk flow features, underlining the complexity of these flows. Observations made here correlate with a qualitative assessment of helicity for different regions of the vessel, presented in Fig. 8 and impact strongly on observed variation of wall loading, as will be discussed later. Figures 4d1–4e1 show the corresponding LNH distributions for two iso-surface values of $\pm 0.8$. Just after systolic peak in Fig. 4d1, the streamwise flow is nearly distinguished with coherent CCW helicity near the left lateral side and CW helicity at the vicinity of right lateral side at the AA and aortic arch. At the end of systole in Fig. 4e1 the helical flow reaches to the DTA. While in diastole, the flow changes its direction in a more random and an irregular fashion with less helicity and vorticity. Ageing within the current range of anatomy variation of aorta slightly alters the coherent helical flow, but it impacts significantly on helicity and helicity intensity, as will be more clarified in the discussion section.

In Figs. 5a–5i, the AV and tangential velocity (TV) components are displayed as area-weighted velocity. Furthermore, Figs. 5j–5l demonstrate the time-averaged, area-weighted axial and tangential velocities, which is labelled as ‘mean velocity’ for simplicity. Comparing the flow rates at Fig. 5, it reveals that, the AV preserves the shape of the inlet flow waveform, irrespective of the age and flow conditions. Moreover, in a cardiac cycle and for a normal and HFF conditions, during the acceleration phase, both velocity components increase with a small phase lag for the TV. But in late systole, once the deceleration phase starts, the axial component diminishes, while the tangential one becomes more significant. Going further, in the diastolic phase, the TVs surpass the AVs and become primary mechanism in flow circulation.

Also, in the normal and HFF (in the HFF the mean flow rate raises up to about 1.5 times) cases and for all the groups, mean values of the axial and tangential velocities in a cardiac cycle are quite close at the AA and ascending aortic arch (AAA), while the sensitivity of TV decreases along the descending aortic arch (DAA) toward the DTA. The latter result is also clarified through the velocity vectors in Fig. 4a. Ageing reduces the velocity inside the aorta; however, the ratio of the axial and tangential velocities remains nearly identical. But, at about the systolic peak and at the AA, where the flow retrogrades, the peak of the TV raises by ageing.

In the LVSD, a decrease in aortic inflow reduces the sensitivity of TV, except in a small portion at the AA, where they are virtually equal. Similar trends can be seen in all the age groups.

### Flow Rate and Pressure Distribution

**Figure 6:**
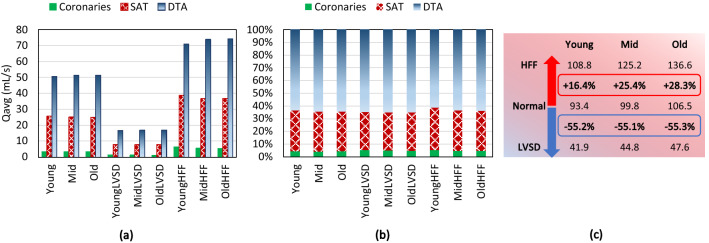
(a) Average flow rate through the coronary arteries, SAT, and DTA, (b) flow percentage through the coronary arteries, SAT, and DTA, and (c) mean pressure in mmHg at the level of aortic root.

Figure 6a shows the average flow rates through coronaries, SAT, and DTA, while Fig. 6b displays the flow percentage through each branch. Comparing different groups, the volume of flow going through each branch is nearly identical for all ages, and the major differences occur once the cardiac function alters. During severe LVSD, the cardiac output diminishes, so the average flow through each branch reduces by 67%. On the contrary, during HFF the heart pumps out more flow to the vascular system, increasing the flow by 46%. Although the average flow magnitude varies at different cardiac functions, the flow percentage through each branch does not change significantly as displayed in Fig. 6b.

Figure 6c shows the mean pressure at the level of aortic root for different age groups and cardiac functions. The results show that the average pressure increases from 93.4 mmHg for the young to 106.5 mmHg for the old group. Furthermore, during the LVSD, a severe flow reduction occurs, therefore, the mean pressure drops significantly up to around 55% for all the ages. Conversely, during the HFF, the mean pressure elevates, while the largest growth is found for the old case with a 28.3% increase.

### Time Averaged Wall Shear Stress (TAWSS), Oscillatory Shear Index (OSI), and ECAP

In clinical context, it has been postulated that for the WSS values less than 0.36 Pa, fatty substances are prone to adhere to the luminal surfaces.^23^ Additionally, high OSI provokes a multi-directional WSS mechanism, which can result in endothelial layer damage and creation of leaky junctions.^30^ Furthermore, the relatively new metric, ECAP^22^ can capture the combined effects of TAWSS and OSI. Therefore, in this section effects of ageing and AF are investigated on the following metrics:

11 $${\text{TAWSS}} = \frac{1}{T}\mathop \smallint \limits_{0}^{T} \left| {\tau_{\text{wall}} } \right|dt$$

12 $${\text{OSI}} = 0.5\left( {1 - \frac{{\frac{1}{T}\left| {\mathop \smallint \nolimits_{0}^{T} \tau_{\text{wall}} dt} \right|}}{{\frac{1}{T}\mathop \smallint \nolimits_{0}^{T} \left| {\tau_{\text{wall}} } \right|dt}}} \right) = 0.5\left( {1 - \frac{{\frac{1}{T}\left| {\mathop \smallint \nolimits_{0}^{T} \tau_{\text{wall}} dt} \right|}}{\text{TAWSS}}} \right)$$

13 $${\text{ECAP}} = \frac{{\text{OSI}}}{{\text{TAWSS}}}$$

During the normal function, the higher velocities in younger cases lead to larger TAWSS magnitudes. On the other hand, as the velocity reduces, more regions are influenced by a higher oscillatory shear. Results show that for the young case, TAWSS is higher, while by arterial dilation in the middle age and old people, even though TAWSS reduces through the entire domain, it still remains significant at the AAA, near the SAT orifices, and at the divider of BCA. In contrast to TAWSS, higher OSI is found for the older people. Given the values of TAWSS and OSI, the maximum ECAP, barely reaches 0.7 Pa^−1^ at the DTA, and occurs for the old case, but it is still below the critical value of 1.4 Pa^−1^^23^,^72^ that causes the vascular damage.

**Figure 7:**
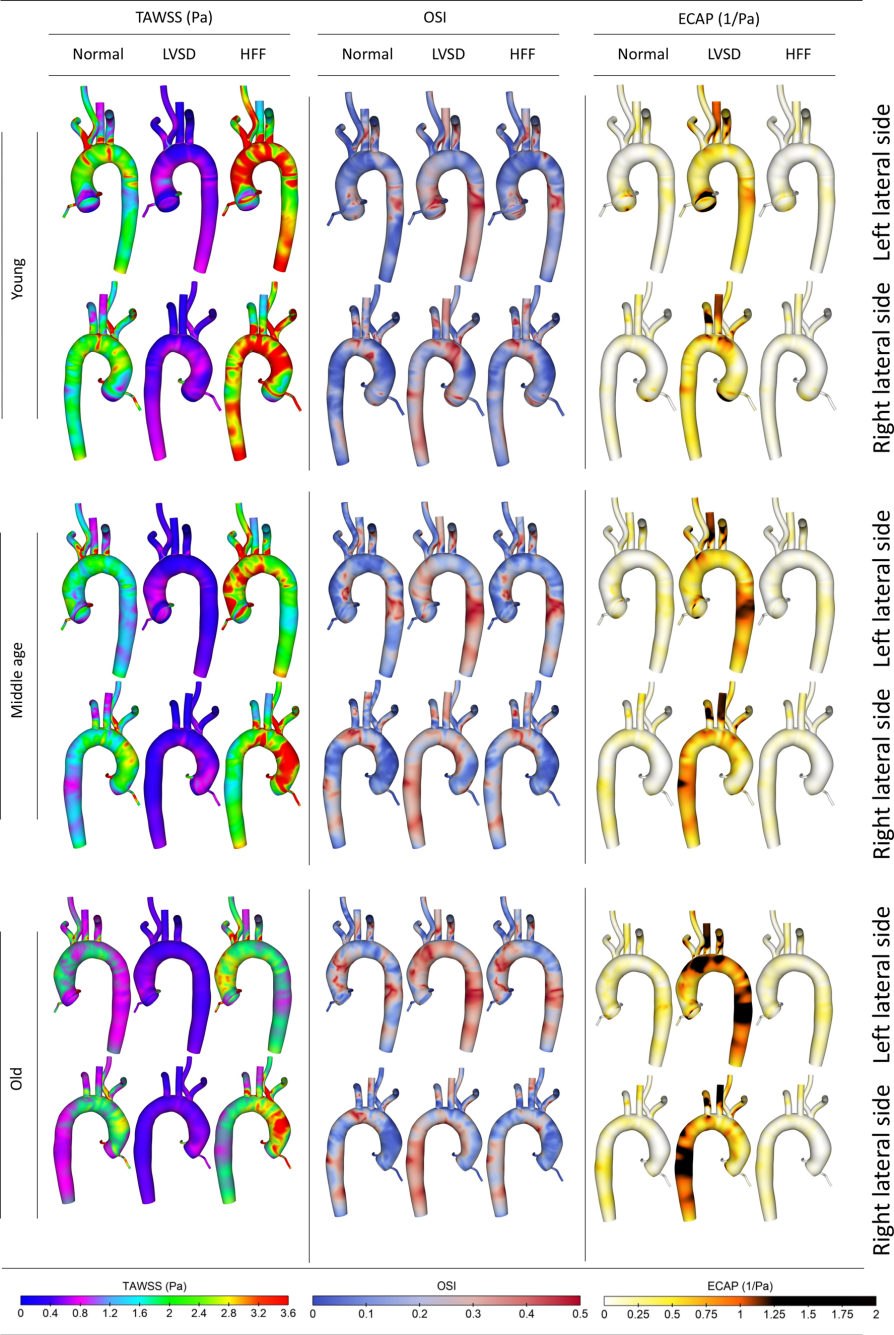
TAWSS, OSI, and ECAP of different age groups for the normal, LVSD, and HFF.

As a result of LVSD, TAWSS diminishes dramatically as shown in Fig. 7, whereas the OSI increases, therefore, the ECAP elevates throughout the entire domain. The results confirm that ageing, due to the morphological changes like aortic dilation and arch unfolding, exacerbates the condition and induces an elevated ECAP to a larger area of SAT, aortic arch and DTA.

HFF can increase the average flow rate through the aorta. Consequently, TAWSS increases dramatically, as shown for the young case in Fig. 7, whereas the middle age and old groups experience a moderate growth. As for the OSI, during HFF, it slightly increases at most of the regions, and through ageing the condition aggravates and higher OSI occurs throughout the domain; hence, the net effect is ECAP reduction. Nevertheless, a much higher TAWSS leads to a much elevated TAWSS gradient (TAWSSG),^19^ which has been recognised as another deteriorating factor of vascular wall.^24^

## Discussion

Velocity components and vortical patterns in a cardiovascular system can be a salient indicator of a cardiac or vascular function.^37^,^69^ Moreover, low TAWSS, high ECAP, and low helicity can diminish atheroprotective phenotypes of endothelial cells.^26^ More specifically, the helicity contributes in an effective blood circulation by preventing excessive energy dissipation, reducing the flow disturbances such as separation and stagnation, reducing LDL infiltration, promoting oxygen uptake, and preserving the luminal surface from platelet and monocyte adhesion.^45^ Therefore, to find a viable correlation between these metrics and their variations due to the ageing and AF, their relative influence needs to be considered and compared.

Irrespective of the age and AF, the results emphasised that, at the AA and AAA the TV has an identical or even higher intensity compared to the AV. Moreover, vortex strength and helicity intensity are higher at these regions. Whereas, in the regions with lower vortex strength, a lower secondary flow and helicity intensity are observed. During a cardiac cycle, at early systole the flow does not form coherent vortical patterns, but as it approaches the systolic peak, a coherent helical flow at the AA appears and moves through the aortic arch and DTA during the deceleration phase (Supplementary Data, Fig. 7); the suggested results are consistent with *in-vivo* measurements.^25^,^53^,^54^ Moreover, through ageing mainly due to the arterial dilation, the magnitude of velocity, vorticity, and helicity reduce, as also confirmed by previous studies.^13^,^25^ Even though the metrics alluded to above change along the aortic conduit from the AA towards the DAA, the main dramatic change occurs for the helicity, where it significantly reduces in the proximity of the DAA towards the end of distal descending thoracic aorta (DDTA). The finding is in accordance with the conclusions drawn by Callaghan *et al*.^13^During the AF, the LVSD and HFF conditions change the haemodynamics differently. In the LVSD, the intensity of TV reduces, and the axial flow comprises the major flow component; also, absence or weak helical flow and the low helicity intensity significantly undermine flow coherence. In the HFF, velocity components do not differ significantly compared to the normal cardiac function, but the helicity increases.

**Figure 8:**
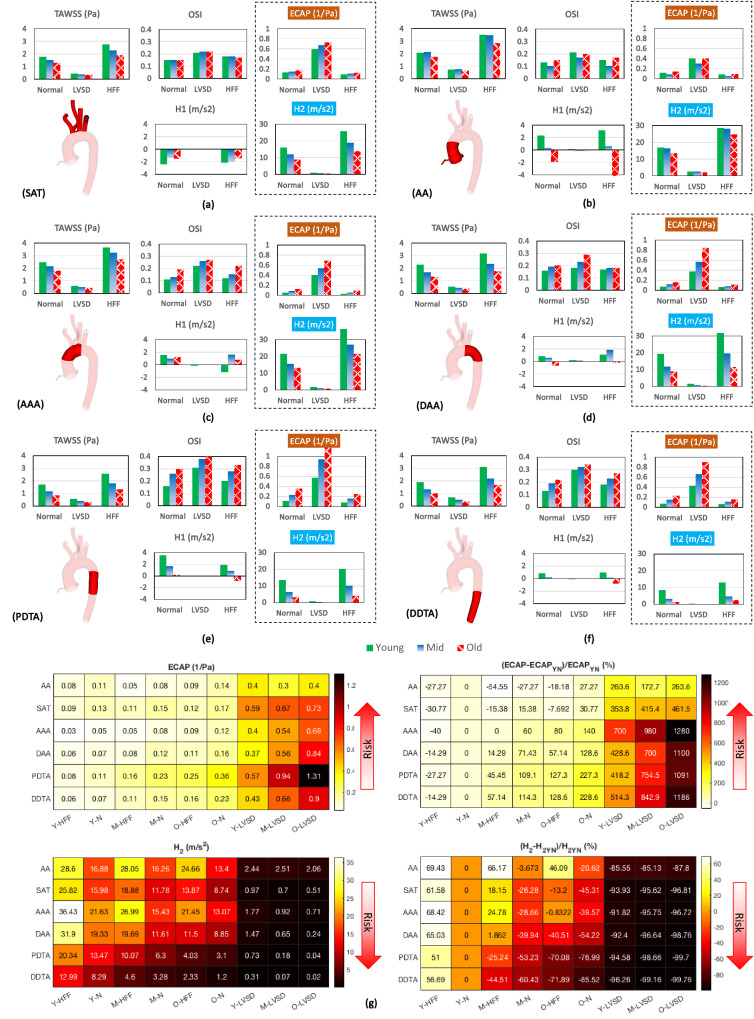
Area-weighted TAWSS (Pa), OSI, ECAP (s^−1^), and time-averaged helicity metrics (*H*_1_, *H*_2_) (m s^2^) versus age and cardiac function for six discrete compartments, including (a) SAT, (b) AA, (c) AAA, (d) DAA, (e) PDTA, and (f) DDTA; (g) heatmaps of ECAP and *H*_2_. ECAP and *H*_2_ increase with respect to the baseline values of the young-normal condition are shown with YN subscript.

Figure 8 summarises TAWSS, OSI, ECAP, helicity, and helicity intensity for different age groups at six defined compartments, namely AA, SAT, AAA, DAA, PDTA, and DDTA to unveil flow characteristics on regional basis.^50^ In Fig. 8, TAWSS, OSI, and ECAP are area-weighted metrics, whereas $H_{1}$ and $H_{2}$ describe mean value of time-averaged helicity within a 3D domain of defined compartments. Furthermore, at the bottom of this figure, the values of ECAP, $H_{2}$, and their increase with respect to the baseline model (young-normal) are presented as heatmaps. The heatmaps demonstrate and rank the riskiest sites and conditions, based on the maximum ECAP and minimum $H_{2}$.

Considering the general impact of ageing, local mean values of TAWSS and helicity decrease, while OSI and ECAP increases, with just an exception at the AA of middle age group, where the OSI and ECAP decreases slightly comparing to the normal case as presented in Fig. 8. The latter might be occurred due to some local geometrical changes, which slightly improved haemodynamic condition. The present findings suggest that during the normal function, the maximum TAWSS reduction is found at the DAA and DTA with about 45% drop in the old case, while the minimum occurs at the AA. On the contrary, the OSI increases at DAA and DTA. Hence, a significant ECAP increase occurs at the aortic posterior regions that reaches around 200% growth, whereas the least growth is observed at AA and SAT. For helicity magnitude, the major drop takes place at the DTA and DAA with around 90% decrease. Considering these changes, it can be concluded that the arterial dilation is the major contributor to the incurred changes. Furthermore, as the dilation is combined with arch unfolding, age-associated changes become more significant at the DAA and DTA.

The net effect of HFF is a significant increase in TAWSS and helicity, and decrease in ECAP with respect to the young-normal case. However, in the middle and old, despite local improvements of flow metrics, the impacts are less pronounced for the middle age and much lesser for the old group as highlighted in Fig. 8g. Moreover, OSI moderately increases due to the HFF, while in contrast to TAWSS, OSI growth is more serious amongst the older people. Given the low ECAP values during the HFF, there might be a slim chance of lipoprotein adhesion to the luminal surface of artery. Nevertheless, in case of an extremely high TAWSS and subsequently a high TAWSSG,^19^,^59^ the configuration and permeability of endothelial cells change, which provoke the leaky junctions and endothelial lesions, and thus make the arterial wall vulnerable to atherogenesis.^17^ On the contrary, during a severe LVSD, ECAP increases and the adhesion mechanism becomes the primary reason of vascular damage. In fact, once the luminal lesion is accompanied by a low WSS, it enhances luminal surface concentration of lipoproteins like albumin and low-density lipoprotein, and consequently infiltration to the vascular wall.^20^,^21^ Moreover, hypertension intensifies penetration in the elderly, with consequences such as intimal thickening, stenosis, and plaque formation,^5^ that increase the risk of ischaemic and thromboembolic strokes.

Comparing ECAP and helicity in Fig. 8, they are somewhat inversely proportional i.e., regional decrease in helicity intensity is accompanied by ECAP increase and vice versa. Therefore, a high helicity intensity strengthens the positive impact of helical flow in reducing luminal surface adhesion/deposition. In addition to ageing, AF affects the helicity by reducing it significantly during the LVSD, while it raises due to the HFF. As shown in Fig. 8g and comparing the results of the ECAP and helicity intensity, the aforementioned points are confirmed.

Therefore, as a summary of the current study the following points are outlined: (i) current meta-analysis confirmed that ageing independently causes aortic dilation and arch unfolding. (ii) high intensity TV at the AA and aortic arch in younger/normal cases are reduced by ageing/AF-LVSD, which is accompanied by occurrence of weaker helicity and vorticity, and higher ECAP, (iii) ageing and AF-LVSD can slightly decrease flow percentage through the SAT, which leads to cerebral hypoperfusion as confirmed in several studies.^1^,^32^,^62^ (iv) ageing due to its morphological changes and elderly hypertension, diminishes the quality of haemodynamic metrics that are mostly reflected as high ECAP and low helicity. Furthermore, once it accompanies with the AF-LVSD, the situation worsens. (v) based on discrete region analysis of the aorta and two measured risk factors (high ECAP and low helicity intensity), it was found that PDTA, DDTA, DAA, AAA are the most vulnerable regions to the vascular damage, respectively. Therefore, the findings suggest that amongst the older people, simultaneous occurrence of mean pressure growth, arterial stiffening, arterial dilation, and aortic arch unfolding increase the risk of arterial wall damage and thrombogenesis. Therefore, the current results suggest that, ageing on its own is a threatening factor for the AF patients, irrespective of previous history of other CVDs or underlaying diseases.

### Limitations

The following considerations could improve the present findings: (1) employing the subject-specific velocity profile at the aortic entrance would improve the accuracy of the results, however, it requires a large dataset of AF patients to evaluate possible variations of inlet velocity, (2) assimilating the wall elasticity for central arteries could improve the results accuracy, while it would require to consider the local variations of elasticity and wall thickness with age in different populations, and (3) While it has been perceived that the coronary resistance changes during AF,^41^ further investigations are required to recognise possible changes in flow characteristics through the aortic branches during AF.

## Electronic supplementary material

Below is the link to the electronic supplementary material.

Electronic supplementary material 1 (PDF 2019 kb)

Electronic supplementary material 2 (M4V 2830 kb)

## Abbreviations

AA: Ascending aorta
AAA: Ascending aortic arch
AF: Atrial fibrillation
AV: Axial velocity
BCA: Brachiocephalic artery
CVD: Cardiovascular disease
CW: Clockwise
CCW: Counter-clockwise
DAA: Descending aortic arch
DDTA: Distal descending thoracic aorta
DTA: Descending thoracic aorta
ECAP: Endothelial cell activation potential
HFF: High frequency fibrillation
LCA: Left coronary artery
LCCA: Left common carotid artery
LSCA: Left subclavian artery
LVSD: Left ventricular systolic dysfunction
LNH: Localised normalised helicity
OSI: Oscillatory shear index
PDTA: Proximal descending thoracic aorta
RCA: Right coronary artery
RCCA: Right common carotid artery
RSCA: Right subclavian artery
SAT: Supra-aortic trunk
TV: Tangential velocity
TAWSS: Time averaged wall shear stress
TAWSSG: Time averaged wall shear stress gradient
