# Supplementary material for: Effects of Ageing on Aortic Circulation During Atrial Fibrillation; a Numerical Study on Different Aortic Morphologies
Source: Ann Biomed Eng. 2021 Mar 2;49(9):2196–213. doi: 10.1007/s10439-021-02744-9 (PMC8455405; doi:10.1007/s10439-021-02744-9)
Supplement: Supplementary file 1 — Electronic supplementary material 1 (PDF 2019 kb) [file 10439_2021_2744_MOESM1_ESM.pdf]

Supplementary Data

1. A survey on variation of the aorta artery geometry by age in healthy cohorts

To understand the geometrical changes of aorta as a result of ageing, a comprehensive survey was carried out on healthy cases without any overt cardiovascular disease. The survey was focused to collect age-associated changes of the main conduit of aorta and the supra-aortic trunk (SAT) configuration.

Table 1. Studies on aorta morphology

Ref	No of Subjects M/F	Total	Age (mean+SD) M/F	Age Range M/F	Ethnicity/Region Population condition
Ref1[1]	64/118	182	46.9±14.6/50±16.4	-	Egypt WRF
Ref2[2]	73/50	123	53±19	14-92	France WRF
Ref3[3]	29/22	51	62±14	34-88	France WRF (suspected to aortic dissection)
Ref4[4]	45/55	100	45±15/48±17	20-84	US WRF
Ref5[5]	Japanese:115/117 American:9/15	256	Japanese: 54±15 American: 43±18	19-79	Japan & US WRF
Ref6[6]	31/30	61	51.4±16.6/49.9±17.9	30-82/18-77	France WRF
Ref7[7]	101/94	195	57±20/56±20	20-96	US Suspected to some sort of RF
Ref8[8]	AA:1,805/1,147 DA:1,195/736	AA: 2,952 DA: 1,931	-	-	US WRF
Ref9[9]	182/162	344	59±16/56±17	14-86/15-91	France WRF(suspected to hypertension)
Ref10[10]	82/128	210	65(median)/61(median)	23-92	Netherland WRF+RF
Ref11[11]	200/0	200	54±8	31-73	NM WRF
Ref12[12]	59/44	103	51±14	-	US WRF
Ref13[13]	71/86	157	49±17	18-77	UK WRF
Ref14[14]	46/24	70	50.5±15.2/49.6±19.1	19-77/17-89	Germany WRF
Ref15[15]	1,767/1,664	3,431 2,343	49.8±10.7/52.2±9.9	>35Yrs/>40Yrs	US WRF
Ref16[16]	NM	100	NM	NM	Egypt WRF
Ref17[17]	54/65	119	NM	9-75	Sweden WRF
Ref18[18]	69/0	69	42.4±16.5	16-75	Germany WRF

NM: Not Mentioned; **CVD**: Cardio-Vascular Disease; **WRF**: Without Risk Factor; **RF**: Risk Factor

Table 1 displays a summary of the main body of the articles from which the related geometrical specifications of aorta were picked. Table 2 represents the percentage of normal SAT – separate origin of brachiocephalic artery (BCA), left common carotid artery (LCCA) and left subclavian artery (LSCA) – from different ethnicities, genders, and regions across the world. The summary emphasises the prevalence of the SAT standard type worldwide.

Table 2. Studies on different configurations of SAT

Ref		Population (No.)	Ethnicity/country	Percentage of normal SAT type
Jakanani et al	[19]	861	UK	74%
Wang et al	[20]	2370	China	83.8%
Muller et al	[21]	2033	Germany	86.7%
Berko et al	[22]	1000	USA	66.5%
Ergun et al	[23]	1001	Turkey	85.2%
Celikyay et al	[24]	1136	Turkey	74.4%
Karacan et al	[25]	1000	Turkey	79.2%
Vucurevic et al	[26]	1266	Serbia	74.7%
Keet et al	[27]	733	African population	65.2%
Aboulhoda et al	[16]	100	Egypt	65%
Popieluszko et al (review article)	[28]	23,882	Review article; different country	80.9%

One of the main difficulties in collecting geometrical data of aorta from different sources is an inconsistency in naming of different parts and thus the associated dimensions. This issue becomes more challenging when the curvilinear or rectilinear length is reported. Figure 1 exhibits the curvilinear length between defined cross sections reported by different sources.

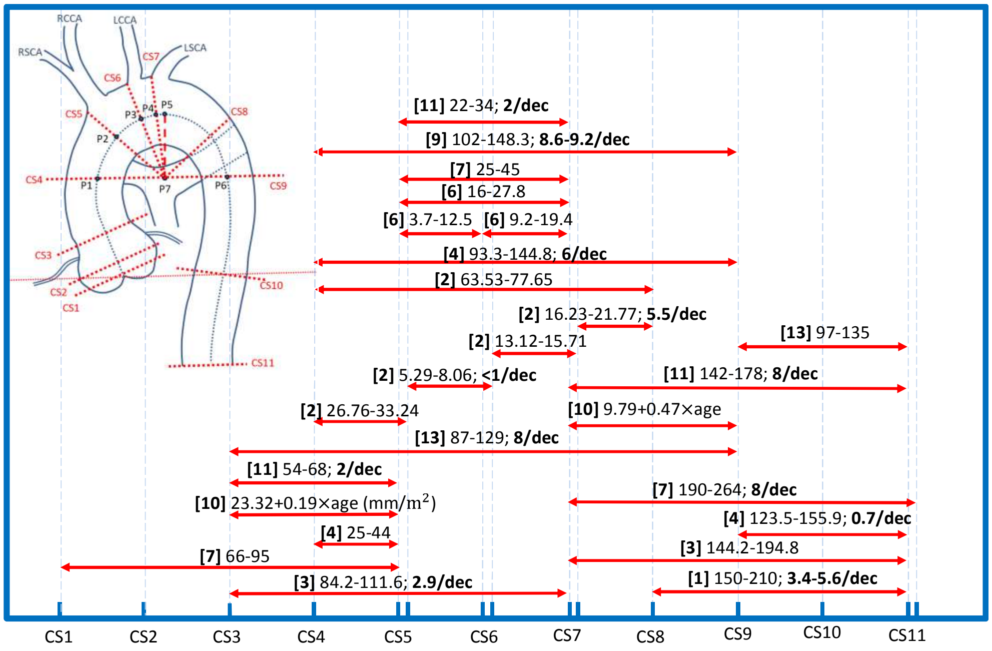

Figure 1. curvilinear length (in mm) as minimum and maximum of average values, and the rate of increase (in mm) per decade of life (dec).

The reported values are minimum and maximum of the average values for various age groups, irrespective of the gender and ethnicity. In fact, the minimum values are associated to the younger people, and the maximum values refer to the older groups. The majority of studies were performed on people in an age range between 20 and 80. In this study, to associate the geometries to different age groups, the collected data are classified as shown in Figure 1 in the main text. Thereafter, starting from the minimum average values for each segment, and based on the mean values of growth per decade of life, new datasets are estimated. Initially, the groups are categorised based on the decade of life as 20-30, 30-40, 40-50, 50-60, 60-70, and 70-80. Then the geometries are constructed for the young, middle age and old groups, who fall between 20-30, 40-60, and above 70, respectively.

2. Three-element Windkessel (RCR WK) model – parameter estimation

To mimic the downstream flow, the RCR WK model is employed. Then, to set a proper lumped-3D coupling. Equation (1) defines the RCR model through a first order ordinary differential equation:

$$\left(1+\frac{R_p}{R_d}\right)Q(t)+R_pC\frac{\mathrm{d}Q(t)}{\mathrm{d}t}=\frac{P(t)}{R_d}+C\frac{\mathrm{d}P(t)}{\mathrm{d}t} \quad (1)$$

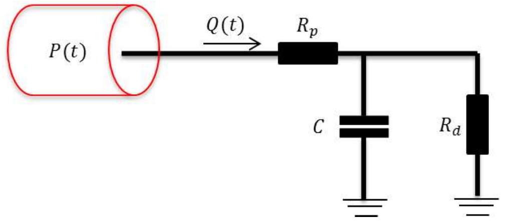

Figure 2. A schematic of three-element Windkessel model

In Eq. (1), R_p and R_d are proximal and distal resistances, respectively, and C is the capacitance of each branch; furthermore, $P(t)$ and $Q(t)$ are the outlet pressure and flow rate, respectively.

To tune the parameter for different age groups, a set of systolic-diastolic pressures was opted for the young, middle age and old groups, which are in a range reported by O'Rourke et al [29]. The mean pressures for the young, middle age and old groups are 93.33, 101.11, and 108.85 mmHg respectively. Furthermore, considering an identical value for the mean flow rate of different ages – 79.2 mL/s – the total resistance was estimated from Eq. (2) as follows:

$$R_{tot}=\frac{P_{avg}}{Q_{avg}} \quad (2)$$

In which mean arterial pressure and flow rates were obtained through Eq. (3) and (4):

$$P_{avg}=\frac{P_{systole}+2P_{diastole}}{3} \quad (3)$$

$$Q_{avg}=\frac{1}{T}\int_0^T Q(t)dt \quad (4)$$

Thereafter, to control a normal blood perfusion through different branches, using the percentage values used by [30] – 1.4% to the LCA, 3.6% to the RCA, 7.5% to each branch of SAT, and 65% to the DTA – the total resistance for each branch is calculated as follows:

$$R_i=\frac{100}{flow\ percentage\ (\%)}R_{tot} \quad (5)$$

To obtain proximal and distal resistance for each branch, they can be estimated by knowing that a proximal resistance (R_p) is around $0.09R_i$ and a distal resistance (R_d) is about $0.91R_i$.

The next step is to find the total capacitance. It has been proven that for the afterload condition, two-element Windkessel model predicts pressure appropriately [31], therefore using the two-element Windkessel model, during the diastole, the pressure can be estimated as:

$$P(t) = ke^{-\frac{t}{R_{total}C_{tot}}} \quad (6)$$

Employing two time points, one just at the end of systole ($\sim\frac{1}{3}t_{cc}$) and another one at the end of diastole, just before the start of systole (t_{cc}), the total capacitance can be obtained through the following equation:

$$C = \frac{2t_{cc}}{3R \times ln(\frac{p_0}{p_1})} \quad (7)$$

In which, P_0 and P_1 are the pressures attributed to $\frac{1}{3}t_{cc}$ and t_{cc}, respectively. In a normal pressure waveform, P_0 is around 90% of systolic pressure. Once the total capacitance is found, the capacitance of each branch can be estimated through Eq. (8):

$$C_i = \frac{flow\ percentage\ (\%)}{100} C_{tot} \quad (8)$$

3. RCR WK implementation

Eq. (1) was discretised implicitly, using first order backward Euler method as follows:

$$P^n = \frac{(R_d\Delta t + R_p\Delta t + CR_pR_d)Q^n - CR_pR_dQ^{n-1} + CR_dP^{n-1}}{CR_d + \Delta t} \quad (9)$$

In Eq. (9), Δt denotes timestep size, while n and n-1 superscripts define two consecutive time points at the current and previous moments, respectively. Thereafter, the discretised model was implemented by writing several User Defined Functions (UDF). The codes were scripted in FORTRAN programming language environment by employing relevant macros for CFX Expression Language (CEL). In particular, eight UDFs were written, one as the master code, which was controlling the seven other codes for each branch. The algorithm of the coding is shown below:

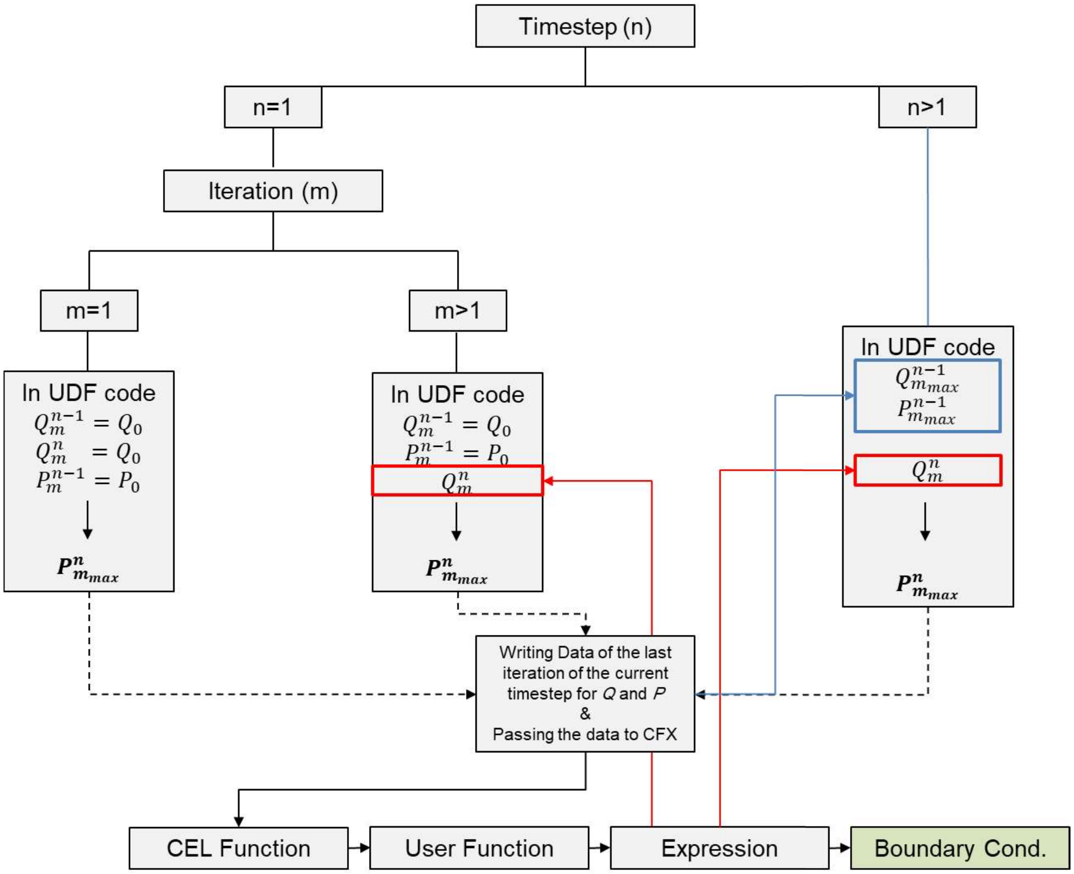

Figure 3. UDF algorithm coupled with ANSYS-CFX

4. Element size and mesh independency

To mesh the computational domain, ANSYS-Meshing toolbox used to obtain a reliable grid network. Given the average inlet velocity, diameter, density and dynamic viscosity and Y^+ = 10, 0.1 of mean aortic diameter was chosen as the total thickness of the prism layer. To have a consistent mesh for different geometries of age classifications, identical element sizes were used for each region as shown below:

Table 3. Element size of each region (mm)

	Coarse	Mid	Fine	Finest
Aorta	1.50	1.00	0.75	0.65
SAT	0.9	0.6	0.45	0.39
Coronaries	0.45	0.3	0.225	0.195

In Table 3, the values under 'Fine' column were chosen as the element size of the final meshes. Furthermore, as presented in Table 4, the prism layer thickness for each region and different age categories are defined.

Table 4. Prism layer thickness (mm)

	Young	Mid	Old
Aorta	2.00	2.11	2.23
SAT	0.85	0.89	0.93
Coronaries	0.32	0.32	0.32

Therefore, as a result between 3.2 and 4.5 million cells were obtained for the young, middle age and old groups, respectively. The result independence from the chosen grid networks are displayed in Figure 5 for seven cross sections.

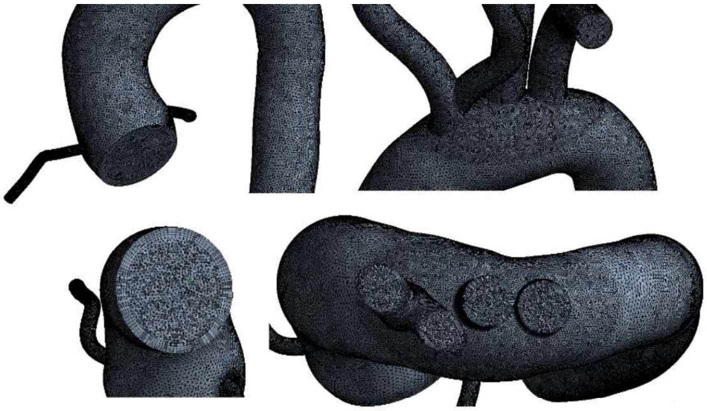

Figure 4. A sample of the meshed computational domain, for the young case

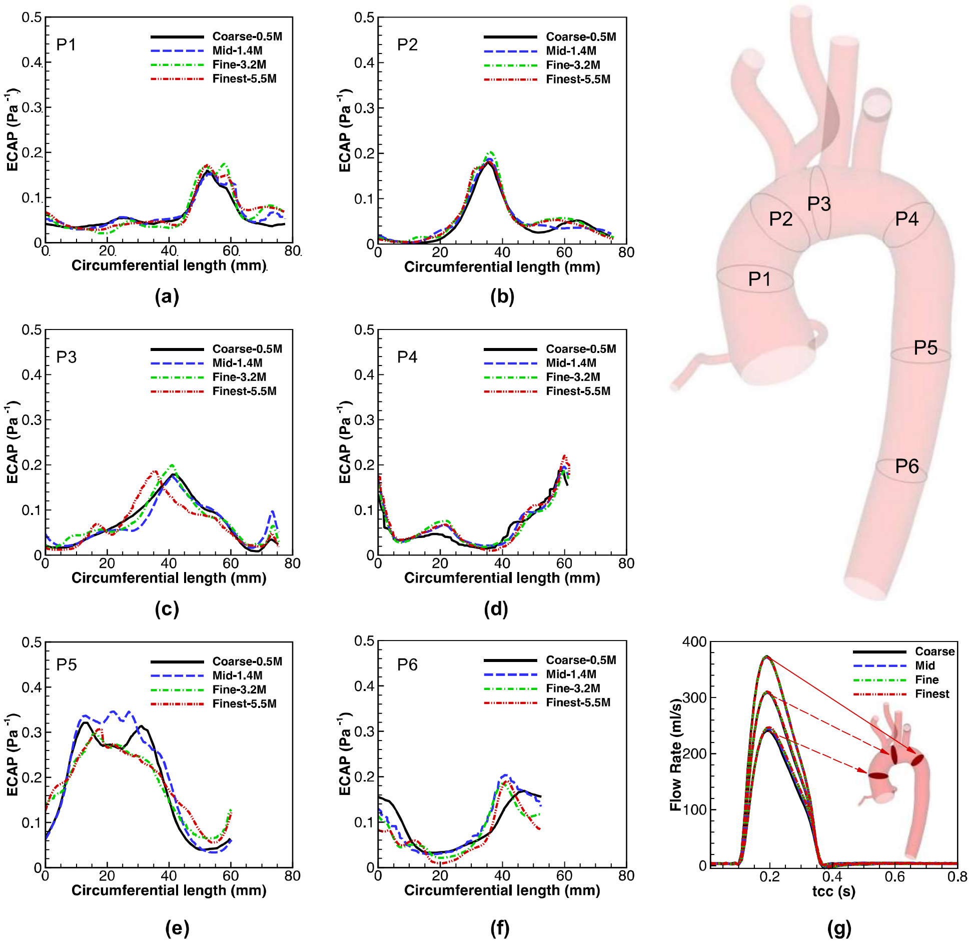

Figure 5. Grid independency result at seven defines cross sections in the main conduit of aorta.

5. Results – flow waveforms along the aorta

Since different age classifications have different geometries, therefore, it is worthy to compare how flow develops from the aortic root towards the descending thoracic aorta. Figure 6 is depicted to show the flow waveforms along the aorta for the normal, LVSD, and HFF. Despite all the morphological differences of aorta at different ages, it is observed that negligible variations can be seen in different phases of a cardiac cycle.

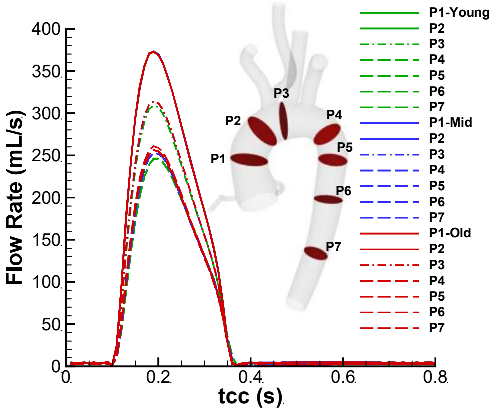

Figure 6. Flow rates along the main conduit of aorta

6. Results – helical flow

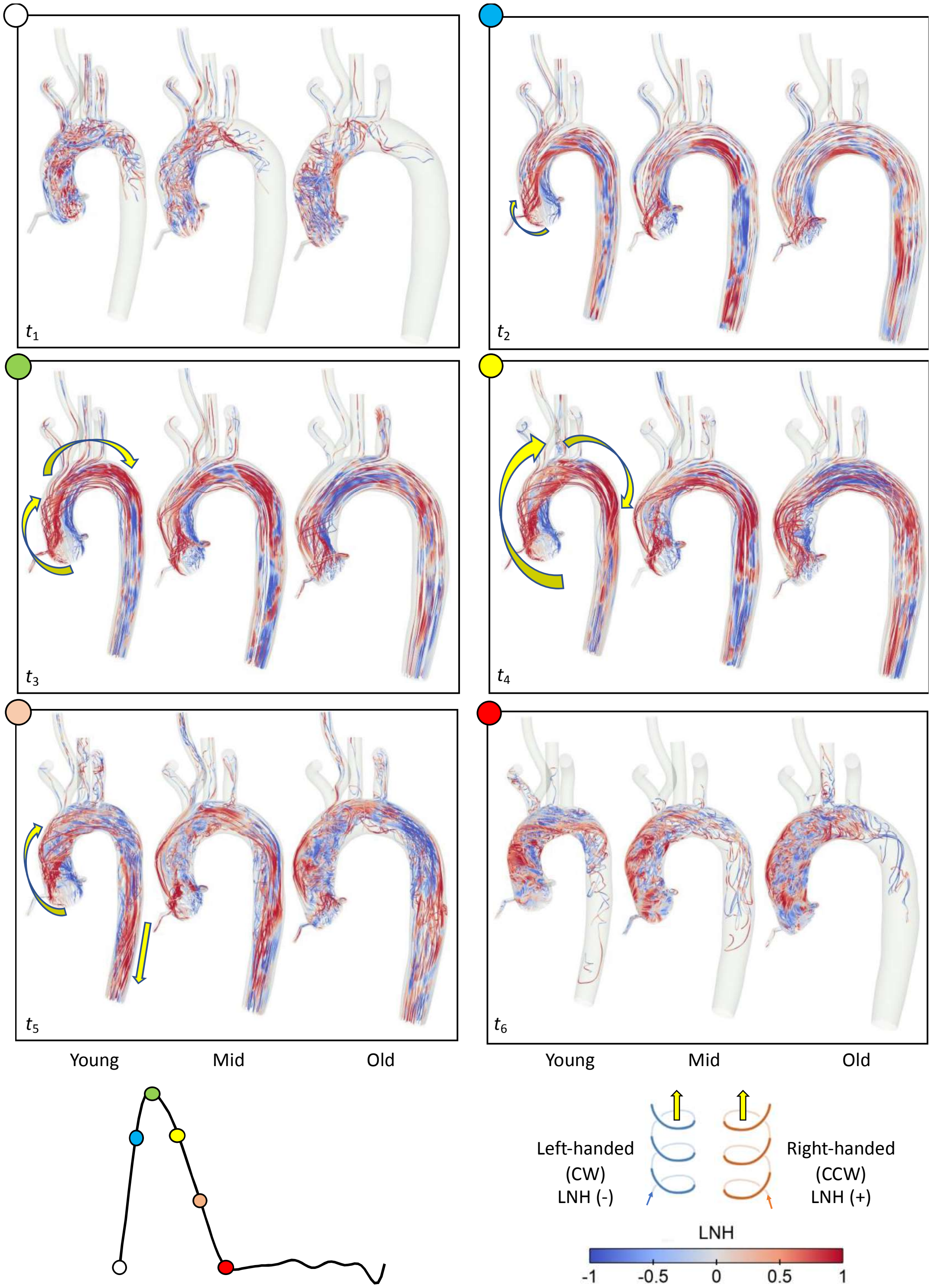

Figure 7. Helical flow in six time-points in a cardiac cycle for the young, middle age, and old groups.

7. References

[1] A. M. Tawfik, D. M. Sobh, B. Gadelhak, H. M. Sobh, and N. M. Batouty, "The effect of age and gender on tortuosity of the descending thoracic Aorta," *Eur. J. Radiol.*, vol. 110, no. September 2018, pp. 54–59, 2019, doi: 10.1016/j.ejrad.2018.11.017.

[2] M. Boufi *et al.*, "Morphological Analysis of Healthy Aortic Arch," *Eur. J. Vasc. Endovasc. Surg.*, vol. 53, no. 5, pp. 663–670, 2017, doi: 10.1016/j.ejvs.2017.02.023.

[3] D. Craiem *et al.*, "Age-related changes of thoracic aorta geometry used to predict the risk for acute type B dissection," *Int. J. Cardiol.*, vol. 228, pp. 654–660, 2017, doi: 10.1016/j.ijcard.2016.11.125.

[4] A. REDEHEUIL *et al.*, "Age-Related Changes in Aortic Arch Geometry : Relationship with Proximal Aortic Function and Left Ventricular Mass and Remodeling," *Am Coll Cardiol.*, vol. 58, no. 12, pp. 1262–1270, 2012, doi: 10.1016/j.jacc.2011.06.012.Age-Related.

[5] J. Sugawara, K. Hayashi, T. Yokoi, and H. Tanaka, "Age-Associated Elongation of the Ascending Aorta in Adults," *JACC Cardiovasc. Imaging*, vol. 1, no. 6, pp. 739–748, 2008, doi: 10.1016/j.jcmg.2008.06.010.

[6] E. Girsowicz *et al.*, "Anatomical Study of Healthy Aortic Arches," *Ann. Vasc. Surg.*, vol. 44, no. November, pp. 179–189, 2017, doi: 10.1016/j.avsg.2017.03.196.

[7] B. Rylski, B. Desjardins, W. Moser, J. E. Bavaria, and R. K. Milewski, "Gender-related changes in aortic geometry throughout life," *Eur. J. Cardio-thoracic Surg.*, vol. 45, no. 5, pp. 805–811, 2014, doi: 10.1093/ejcts/ezt597.

[8] A. Wolak *et al.*, "Aortic Size Assessment by Noncontrast Cardiac Computed Tomography: Normal Limits by Age, Gender, and Body Surface Area," *JACC Cardiovasc. Imaging*, vol. 1, no. 2, pp. 200–209, 2008, doi: 10.1016/j.jcmg.2007.11.005.

[9] N. d'ostrevy *et al.*, "The apex of the aortic arch backshifts with aging," *Surg. Radiol. Anat.*, vol. 39, no. 7, pp. 703–710, 2017, doi: 10.1007/s00276-016-1792-9.

[10] B. P. Adriaans *et al.*, "Aortic elongation part I: The normal aortic ageing process," *Heart*, vol. 104, no. 21, pp. 1772–1777, 2018, doi: 10.1136/heartjnl-2017-312866.

[11] D. Craiem *et al.*, "Aging impact on thoracic aorta 3D morphometry in intermediate-risk subjects: Looking beyond coronary arteries with non-contrast cardiac CT," *Ann. Biomed. Eng.*, vol. 40, no. 5, pp. 1028–1038, 2012, doi: 10.1007/s10439-011-0487-y.

[12] F. Y. Lin *et al.*, "Assessment of the thoracic aorta by multidetector computed tomography: Age- and sex-specific reference values in adults without evident cardiovascular disease," *J. Cardiovasc. Comput. Tomogr.*, vol. 2, no. 5, pp. 298–308, 2008, doi: 10.1016/j.jcct.2008.08.002.

[13] S. S. Hickson *et al.*, "The relationship of age with regional aortic stiffness and diameter," *JACC Cardiovasc. Imaging*, vol. 3, no. 12, pp. 1247–1255, 2010, doi: 10.1016/j.jcmg.2010.09.016.

[14] A. Hager *et al.*, "Diameters of the thoracic aorta throughout life as measured with helical computed tomography," *J. Thorac. Cardiovasc. Surg.*, vol. 123, no. 6, pp. 1060–1066, 2002, doi: 10.1067/mtc.2002.122310.

[15] I. S. Rogers *et al.*, "Distribution, determinants, and normal reference values of thoracic and abdominal aortic diameters by computed tomography (from the

framingham heart study)," *Am. J. Cardiol.*, vol. 111, no. 10, pp. 1510–1516, 2013, doi: 10.1016/j.amjcard.2013.01.306.

[16] B. E. Aboulhoda, R. K. Ahmed, and A. S. Awad, "Clinically-relevant morphometric parameters and anatomical variations of the aortic arch branching pattern," *Surg. Radiol. Anat.*, vol. 41, no. 7, pp. 731–744, 2019, doi: 10.1007/s00276-019-02215-w.

[17] F. Hansen, P. Mangell, B. Sonesson, and T. Länne, "Diameter and compliance in the human common carotid artery - variations with age and sex," *Ultrasound Med. Biol.*, vol. 21, no. 1, pp. 1–9, 1995, doi: 10.1016/0301-5629(94)00090-5.

[18] A. Schmidt-Trucksäss *et al.*, "Structural, Functional, and Hemodynamic Changes of the Common Carotid Artery With Age in Male Subjects," *Arterioscler. Thromb. Vasc. Biol.*, vol. 19, no. 4, pp. 1091–1097, Apr. 1999, doi: 10.1161/01.ATV.19.4.1091.

[19] G. C. Jakanani and W. Adair, "Frequency of variations in aortic arch anatomy depicted on multidetector CT," *Clin. Radiol.*, vol. 65, no. 6, pp. 481–487, 2010, doi: 10.1016/j.crad.2010.02.003.

[20] L. Wang, J. Zhang, and S. Xin, "Morphologic features of the aortic arch and its branches in the adult Chinese population," *J. Vasc. Surg.*, vol. 64, no. 6, pp. 1602-1608.e1, 2016, doi: 10.1016/j.jvs.2016.05.092.

[21] M. Müller *et al.*, "Variations of the aortic arch - A study on the most common branching patterns," *Acta radiol.*, vol. 52, no. 7, pp. 738–742, 2011, doi: 10.1258/ar.2011.110013.

[22] N. S. Berko, V. R. Jain, A. Godelman, E. G. Stein, S. Ghosh, and L. B. Haramati, "Variants and anomalies of thoracic vasculature on computed tomographic angiography in adults," *J. Comput. Assist. Tomogr.*, vol. 33, no. 4, pp. 523–528, 2009, doi: 10.1097/RCT.0b013e3181888343.

[23] E. Ergun, B. Şimşek, P. N. Koşar, B. K. Yilmaz, and A. T. Turgut, "Anatomical variations in branching pattern of arcus aorta: 64-slice CTA appearance," *Surg. Radiol. Anat.*, vol. 35, no. 6, pp. 503–509, 2013, doi: 10.1007/s00276-012-1063-3.

[24] Z. R. Y. Celikyay, A. E. Koner, F. Celikyay, C. Deniz, B. Acu, and M. M. Firat, "Frequency and imaging findings of variations in human aortic arch anatomy based on multidetector computed tomography data," *Clin. Imaging*, vol. 37, no. 6, pp. 1011–1019, 2013, doi: 10.1016/j.clinimag.2013.07.008.

[25] A. Karacan, A. Türkvatan, and K. Karacan, "Anatomical variations of aortic arch branching: Evaluation with computed tomographic angiography," *Cardiol. Young*, vol. 24, no. 3, pp. 485–493, 2014, doi: 10.1017/S1047951113000656.

[26] G. Vučurević *et al.*, "Anatomy and radiology of the variations of aortic arch branches in 1,266 patients," *Folia Morphol.*, vol. 72, no. 2, pp. 113–122, 2013, doi: 10.5603/FM.2013.0019.

[27] K. Keet, G. Gunston, and R. Alexander, "Variations in the branching pattern of the aortic arch: An African perspective," *Eur. J. Anat.*, vol. 23, no. 2, pp. 91–102, 2019.

[28] P. Popieluszko *et al.*, "A systematic review and meta-analysis of variations in branching patterns of the adult aortic arch," *J. Vasc. Surg.*, vol. 68, no. 1, pp. 298-306.e10, 2018, doi: 10.1016/j.jvs.2017.06.097.

[29] M. F. O'Rourke and W. W. Nichols, "Aortic diameter, aortic stiffness, and wave reflection increase with age and isolated systolic hypertension," *Hypertension*, vol. 45, no. 4 SUPPL., pp. 652–658, 2005, doi: 10.1161/01.HYP.0000153793.84859.b8.

[30] A. C. Benim, A. Nahavandi, A. Assmann, D. Schubert, P. Feindt, and S. H. Suh, "Simulation of blood flow in human aorta with emphasis on outlet boundary conditions," *Appl. Math. Model.*, vol. 35, no. 7, pp. 3175–3188, Jul. 2011, doi: 10.1016/j.apm.2010.12.022.
[31] Y. Shi, P. Lawford, and R. Hose, "Review of Zero-D and 1-D Models of Blood Flow in the Cardiovascular System," *Biomed. Eng. Online*, vol. 10, no. 1, p. 33, 2011, doi: 10.1186/1475-925X-10-33.
[32] M. A. Simaan, A. Ferreira, S. Chen, J. F. Antaki, and D. G. Galati, "A dynamical state space representation and performance analysis of a feedback-controlled rotary left ventricular assist device," *IEEE Trans. Control Syst. Technol.*, vol. 17, no. 1, pp. 15–28, 2009, doi: 10.1109/TCST.2008.912123.
[33] N. Stergiopulos, J. J. Meister, and N. Westerhof, "Determinants of stroke volume and systolic and diastolic aortic pressure.," *Am. J. Physiol.*, vol. 270, no. 6 Pt 2, pp. H2050-9, 1996, doi: 10.1152/ajpheart.1996.270.6.H2050.
[34] S. Scarsoglio, A. Guala, C. Camporeale, and L. Ridolfi, "Impact of atrial fibrillation on the cardiovascular system through a lumped-parameter approach," *Med. Biol. Eng. Comput.*, vol. 52, no. 11, pp. 905–920, 2014, doi: 10.1007/s11517-014-1192-4.
